# Performance of Modified Cement-Based Slurry Incorporation with Multi-Walled Carbon Nanotubes (MWCNTs), Polycarboxylate Ether Superplasticizer (PCE) and Hydroxypropyl Methylcellulose (HPMC) Under High-Temperature

**DOI:** 10.3390/ma19132912

**Published:** 2026-07-07

**Authors:** Xianjie Weng, Yuhao Song, Wu Zeng, Zhou Lv, Xing Liu, Lianzhen Zhang, Hao Tong

**Affiliations:** 1 School of Civil Engineering, Chongqing University, Chongqing 400045, China; 13054984932@163.com; 2 College of Pipeline and Civil Engineering, China University of Petroleum, Qingdao 266580, China; 15964190211@163.com (Y.S.); 15079316585@163.com (H.T.); 3 Jiangxi Communications Investment Group Co., Ltd., Nanchang 330108, China; 15954760091@163.com (W.Z.); hyw24060023@163.com (Z.L.); limingchenupc@163.com (X.L.)

**Keywords:** high temperature, dynamic water, multi-walled carbon nanotubes (MWCNTs), polycarboxylate ether superplasticizer (PCE), hydroxypropyl methylcellulose (HPMC)

## Abstract

**Highlights:**

- A synergistic additive system (MWCNTs, PCE, HPMC) is proposed to overcome the degradation of cement grout under high-temperature and dynamic water scouring.
- Reinforcement mechanism at 80 °C elucidated: 0.32 wt% MWCNTs inhibit late-stage ettringite formation, provide nucleation/encapsulation sites, and bridge hydration products.
- 28-day compressive strength increased by 20% (up to 26.4 MPa) with only 0.32 wt% MWCNTs at 80 °C.
- A dynamic water retention ratio is introduced to realistically simulate inrush conditions, enabling quantitative evaluation of grout performance under flowing water.
- Optimal formulation (0.3% PCE + 0.2% HPMC) improves fluidity by ~8% and dynamic retention by up to 38% at 1.0 m/s flow velocity, fully supported by XRD, TG, and SEM.

**Abstract:**

Cement slurry is a staple grouting agent, yet its properties can weaken when exposed to heat. Studying grouting materials for use in high-temperature tunnels is therefore a matter of considerable importance. To enhance the applicability of cement-based slurry in high-temperature tunnels, multi-walled carbon nanotubes (MWCNTs), polycarboxylate ether superplasticizer (PCE), and hydroxypropyl methylcellulose (HPMC) were added to improve their performance at elevated temperatures. Various experimental methods were employed to investigate the properties of the modified slurry at different temperatures, including flowability, setting time, compressive strength, and dynamic water retention ratio. Additionally, X-ray diffraction (XRD), thermogravimetric analysis (TG), and scanning electron microscopy (SEM) were used to study the effects of temperature on hardened slurry. Experimental results indicate that the optimal MWCNTs content is 0.32%. At this content, the compressive strength of the hardened slurry after 28 days of curing at 80 °C increases by approximately 20%, reaching 26.4 MPa. PCE improves the fluidity of the slurry, while HPMC enhances its water dynamic water retention ratio. The optimal proportion was found to be 0.3% PCE and 0.2% HPMC. At this ratio, the fluidity of the slurry increased by about 8%, reaching approximately 17.7 cm; the dynamic water retention ratios of 0.8 m/s and 1.0 m/s improved by approximately 22% and 38%, respectively, achieving 35.8% and 18.1%. Furthermore, multi-walled carbon nanotubes significantly enhance the compressive strength of the hardened slurry primarily by suppressing the formation of ettringite during the later stages of hydration, as well as by providing nucleation sites, encapsulating hydration products, and bridging hydration product clusters within the microstructure. This investigation lays a theoretical groundwork for formulating and choosing grouting materials suited to high-temperature tunnel environments.

## 1. Introduction

As underground space development extends to greater depths, a number of deep-buried tunnels subjected to elevated temperatures have been encountered, including the Changdu Tunnel (73 °C), the Zila Mountain Tunnel (60 °C), the Bangda Tunnel (64 °C), and the Jianyuan Expressway Nige Tunnel (88 °C) [1]. Thermal inrush events from fissures are commonly driven by a combination of elevated water pressure within the fractures and vigorous geological activity [2].

It has been well established that grouting serves as a viable approach for managing water inrush incidents [3,4,5,6]. However, conventional grouting materials exhibit some performance limitations at high-temperature conditions [7,8]. For example, in the hydrodynamic grouting environment, ordinary cement slurry is easily washed and diluted by water flow after being injected into the water-inrush formation, resulting in substantial deterioration of its working and mechanical properties, which seriously affects the grouting reinforcement and plugging effect. B. Lothenbach [9] discovered that the early strength of ordinary Portland cement grout was improved, while later strength was significantly reduced, within the temperature range of 5–50 °C. L. Fan [10] observed that the compressive strength of cement stone at 28 days of curing decreased with increasing temperature. In contrast, Li [11] studied the strength of ordinary Portland cement grout cured at 80 °C for 28 days and found that it was higher than that cured at 20 °C. As found above, the influence of temperature on cement grout strength is intricate and depends on multiple factors. Zhang [12] verified that fly ash and 300-mesh silica can improve various properties of cement slurry under high-temperature conditions. Nevertheless, the fluidity, compressive strength and dynamic water retention ratio of this cement-based slurry are still unsatisfactory, and further performance enhancement is required.

Research on the efficient application of nanomaterials in construction engineering has become a prominent research topic. Multi-walled carbon nanotubes (MWCNTs) are hollow nano-sized tubular carbon materials rolled up by multiple layers of graphene sheets. They possess the intrinsic properties of carbon materials as well as the size effect of nanomaterials, and their addition in a small content can enhance the performance of matrix materials. Existing studies have demonstrated that MWCNTs improve cement properties mainly in two aspects: first, they fill pores at the microstructural level; second, they act as nucleating agents to accelerate the hydration reaction of cement. Zhao [13] found that MWCNTs can promote cement hydration and the formation of C-S-H gel. Li [14] investigated the effect of MWCNTs on cement hydration. Microscopic analysis of cement slurry incorporated with MWCNTs revealed that MWCNTs serve as nucleation sites, facilitate the production of calcium hydroxide (CH) hydration products, modify the crystal plane size of CH crystals, and thereby increase the strength of cement slurry. Through analysis, Chen [15] established a porosity-based method for characterizing the dispersion of MWCNTs, and demonstrated that their uniform dispersion facilitates hydration, refines the pore structure, and consequently enhances concrete performance, whereas the agglomeration of MWCNTs degrades the microstructure and results in performance degradation. Li [16] studied the influence of MWCNT content (0.1~0.5 wt%) on the early-age hydration process (1 day) of cement-based slurry. The results indicated that MWCNTs exert no effect on the type of early-age cement hydration products. Liu [17] investigated the effect of using multi-walled carbon nanotubes (MWCNTs) of three different diameters, dispersed by polyvinylpyrrolidone (PVP), to reinforce ultrafine cement grouting material on the rheological properties of the grouting material. Guan [18] explored the effect of MWCNTs content on the strength of cement slurry. The test results show that when the MWCNTs content is 0.5%, the 14-day compressive strength of cement slurry increases by 25.6% compared with the blank group.

When grouting materials are used to improve the strength of cement-based slurry, they inevitably impair other workability properties, such as reduced fluidity and prolonged setting time. Accordingly, additional admixtures are required to optimize these performances. Polycarboxylate superplasticizer (PCE) is a commonly used water-reducing agent. It features a high water reduction rate, low slump loss, environmental friendliness, and excellent compatibility with cementitious materials, and has been widely applied in the concrete industry [19]. Wan [20] and Zhang [21] incorporated PCE into concrete and observed a remarkable improvement in fluidity. Based on experimental results, they recommended the optimal PCE content s of 0.3% and 0.4% respectively. Luis added PCE to ordinary Portland cement and concluded that PCE effectively enhanced slurry fluidity while exerting little influence on the porosity of hardened slurry [22]. Hydroxypropyl methylcellulose (HPMC) is a typical non-ionic cellulose ether widely used in construction engineering. With superior water retention and thickening properties, it is extensively applied in self-leveling floor mortar and self-compacting concrete, which greatly improves material performance and construction efficiency [23]. Flocculants, also acting as defoamers, possess favorable chemical stability and good compatibility with other admixtures.

Gu [24] investigated the properties of concrete blended with HPMC. The results showed that HPMC increased compressive strength and viscosity and reduced porosity, but it would decrease fluidity. Ding [25] found that HPMC could improve the fluidity and plasticity of cement slurry and exert a retarding effect. The retarding effect became more pronounced with the increase in HPMC content. Chen [26] stated that HPMC can form a colloidal film in water and build a network structure via cross-linking. This structure adheres to the surface of Portland cement or sand particles, hinders water migration inside the slurry, and thereby enhances the water retention capacity.

In this study, MWCNTs, PCE, and HPMC were incorporated into the cement-fly ash-300-mesh silica (C-F-S) composite slurry to further enhance its performance at high temperatures. The improvement effects of MWCNTs, PCE and HPMC on the high-temperature properties of C-F-S slurry were evaluated by taking fluidity, setting time, anti-dispersion performance in flowing water and compressive strength at different temperatures as evaluation indicators. Meanwhile, X-ray diffraction (XRD), thermogravimetry-derivative thermogravimetry (TG-DTG) and scanning electron microscopy (SEM) were adopted to analyze the variations in hydration products and microstructure of the slurry under high-temperature conditions. The modification mechanism of MWCNTs on the high-temperature performance of the slurry was revealed. The novelty of this research lies in developing a cement-fly ash-300-mesh silica sand (C-F-S) composite grouting material by adding a series of additives. This material has the advantages of high strength, strong fluidity, and good dynamic water retention performance in high-temperature environments. This material provides an effective solution for grouting technology in high-temperature flowing water environments.

## 2. Materials and Methods

### 2.1. Raw Materials

**Figure 1:**
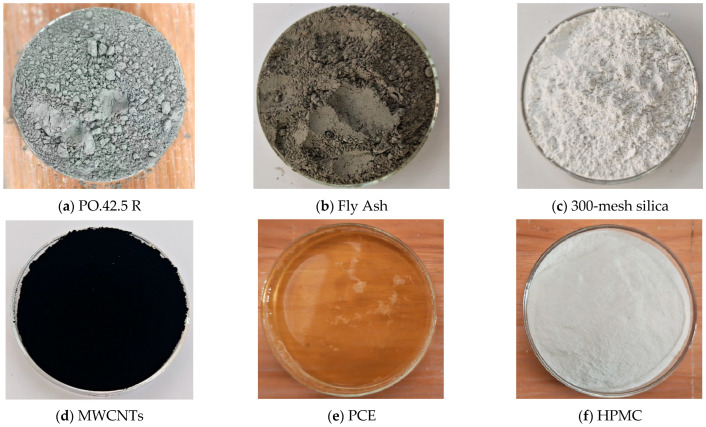
Raw materials: (**a**) PO.42.5 R; (**b**) Fly Ash; (**c**) 300-mesh silica; (**d**) MWCNTs; (**e**) PCE; (**f**) HPMC.

For the test, the cement employed was ordinary Portland cement of strength class 42.5R, meeting the national standard GB 175-2023 [27]. The fly ash was obtained from Gongyi Borun Refractory Materials Co., Ltd. Zhengzhou, China, and its technical indicators satisfied the requirements of GB/T 1596-2017 [28]. Silicon dioxide powder (300 mesh, 48 μm) was provided by Henan Yixinglong Environmental Protection Technology Co., Ltd., Zhengzhou, China, and its performance conformed to GB/T 1347-2008 [29]. The chemical compositions of the raw materials are presented in Table 1, and their photographs are shown in Figure 1.

The MWCNTs adopted in this study were manufactured by Shenzhen Suiheng Technology Co., Ltd., Shenzhen, China. Their technical parameters are as follows: purity > 97%, inner diameter 3–5 nm, outer diameter 8–15 nm, length 3–12 μm, specific surface area ≥ 270 m^2^/kg, bulk density 0.07 g/cm^3^, particle size ≤ 7 μm. The product was synthesized via the chemical vapor deposition (CVD) method. Its production process and performance indicators comply with the national standard Nanotechnology Characterization of multi-walled carbon nanotubes (GB/T 33243-2016) [30]. The PCE was supplied by Sichuan Dongrun Baisheng New Materials Co., Ltd., Chengdu, China, with a solid content of 40.2%, pH value of 6.5, and density of 1.027 g/cm^3^, and its water reduction rate reaches 27%. HPMC was produced by Weihui Tensheng Trading Co., Ltd., Shenzhen, China, which is a grade with a viscosity of 100,000 mPa·s. The loss on drying was determined, and the pH of a 1% aqueous solution at 25 °C fell within the range of 4.0 to 8.0. Both admixtures complied with the national standards GB 8076-2008 (concrete admixtures) [31] and GB/T 8077-2023 (test methods for uniformity of concrete admixtures) [32]. Their physical appearance is presented in Figure 1.

### 2.2. Experimental Scheme

The evaluation of the modified cement-based grout mainly covered bleeding rate, flowability, setting time, compressive strength, and resistance to washing out under dynamic water conditions.

Considering that field temperatures can reach 88 °C, as recorded for the Nige Tunnel along the Jianyuan Expressway [2], the highest experimental temperature was set at 95 °C. Room temperature (20 °C) was adopted as the lowest point to provide a baseline for comparison. The tests were therefore carried out at six temperature levels: 20, 35, 50, 65, 80, and 95 °C. All materials and beakers were preheated in a water bath, and a thermometer was used to track the temperature. The mixture was stirred constantly to make up for heat lost during the mixing process.

To determine the optimal content of MWCNTs, PCE and HPMC, four MWCNT contents were designed as 0.24%, 0.32%, 0.40% and 0.48% [15]. The selected PCE contents were 0.10%, 0.30% and 0.50% [20], while the HPMC contents were set at 0.10%, 0.15% and 0.20% [24]. The compressive strength of the modified cement-based grout was evaluated for various mix proportions and curing times (3, 7, 14, and 28 days). Its performance characteristics, including flowability, setting time, and resistance to washout under flowing water, were also examined. For the dynamic water retention test, flow velocities of 0.8 m/s and 1.0 m/s were selected [33]. The experimental scheme is presented in Table 2. When using MWCNTs, various dispersion methods such as physical and ultrasonic methods can be employed to enhance their dispersion in the slurry. However, this paper believes that some physical methods, due to their excessive intensity, may damage the structure of MWCNTs, resulting in a decline or even loss of their performance. Moreover, ultrasonic methods and other means are costly and not suitable for operation in the engineering site. Therefore, in this section, a simple stirrer is selected for stirring to disperse MWCNTs.

### 2.3. Test Methods

#### 2.3.1. Fluidity Test

The fluidity test was performed over a temperature range of 20–95 °C according to the Chinese Standard Test methods for the uniformity of concrete admixtures (GB/T 8077-2023) [32]. A 1000 mm × 1000 mm × 5 mm heating pad was positioned underneath the glass plate to control the plate temperature and to offset the heat loss of the slurry during the test. The required materials and water are weighed. The dry constituents are first mixed and heated in a water bath, while the temperature is monitored in real time with a thermometer. Once the target temperature is reached, water is added, and the mixture is stirred into a slurry while being maintained in the water bath to preserve the temperature. During stirring, the beaker is immersed as fully as possible in the water bath—taking care to prevent bath water from entering the beaker—so as to compensate for the heat loss during mixing; the slurry temperature is continuously monitored with a thermometer throughout this process. Right after mixing, the slurry is quickly poured into a truncated cone mold and leveled off at the top surface. The mold is then lifted straight up to allow the slurry to slump and spread freely. Once the slurry stops flowing, the spread diameter in one direction is measured several times, and the average of these readings is taken as the final result.

#### 2.3.2. Setting Time Test

The setting time of the slurry was determined following the Chinese standard that covers test methods for normal consistency water requirement, setting time, and soundness (GB 1346-2024) [34]. The slurry was poured into a truncated cone mold, which was then placed in a constant-temperature water bath. The water level was kept below the top surface of the mold to avoid any contact between the water and the slurry. To minimize the adverse effects of temperature fluctuations on the measurement accuracy of the setting time, the entire operation process, from removing the truncated cone mold specimen to completing the Vicat apparatus measurement, was strictly controlled within 10 s. This kept the temperature loss of the slurry within a tolerable range, thus allowing for accurate measurement of its setting time under high-temperature conditions.

#### 2.3.3. Compressive Strength Test

In field grouting operations within high-temperature, water-saturated fractured rock, the grout is batched at ambient temperature and then injected into hot fractures, where it warms up to the rock temperature as it travels. To reproduce this real-world scenario, the slurry was mixed at room temperature and subsequently cured at elevated temperatures.

**Figure 2:**
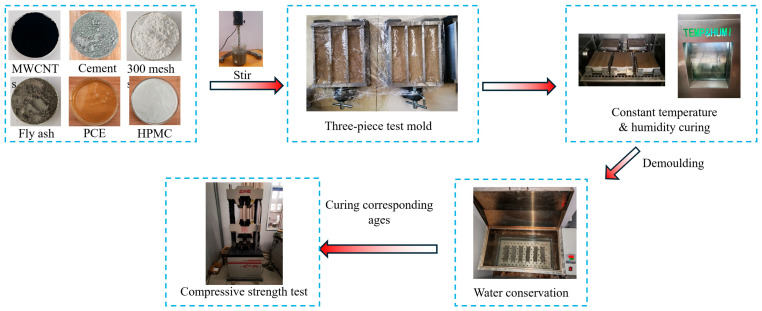
Molding process for slurry samples.

Since the rock fractures are saturated with water, the slurry ought to be kept in a constant-temperature water bath as far as possible during the test. However, directly curing the slurry samples in a water bath would cause severe mixing with water, thereby reducing the W/C ratio and reducing the strength of the slurry. Therefore, in the actual testing process, the slurry was first cast into constant-temperature and constant-humidity molds (40 mm × 40 mm × 160 mm) until setting was complete. Once demolded, the slurry specimens were transferred to a thermostatic water bath and cured for the specified duration. Compressive strength was determined according to the Chinese standard test method for cement mortar strength (GB/T 17671-2021) [35]. Figure 2 illustrates the detailed molding procedure for the slurry specimens.

#### 2.3.4. Grout Retention Ratio Test Under Dynamic Water

**Figure 3:**
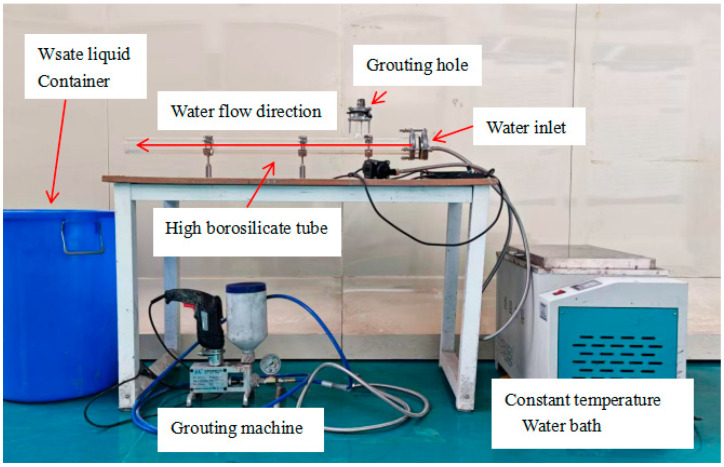
Testing system for the grout retention ratio under dynamic water.

A purpose-built dynamic water scouring simulator (Figure 3) was used to evaluate the slurry’s resistance to washout under flowing water. The test rig consisted of four parts: a high-temperature water flow generation module, a water conveyance channel module, a grouting module, and a waste liquid collection module. The high-temperature flow was generated by a constant-temperature water bath and a pump. A 1 m borosilicate glass tube served as the conveyance channel to ensure minimal deformation at elevated temperatures. Once the water reached the target temperature, the pump delivered it into the channel. The grouting module comprised a grouting machine and a connected pipeline. The injection pressure was kept at 50 kPa, and the grouting hole (2.5 cm in diameter) was positioned 25 cm from the water inlet.

The prepared slurry was filled into the grouting machine, and the combined weight of the machine and slurry was taken as m_1_. To begin the test, the water pump was started and set to the predetermined scouring flow rate. Once the water flow became steady, the grouting machine was activated and kept running until all the slurry had been injected, after which it was turned off. Throughout the test, a portion of the slurry was swept into the waste liquid container by the flowing water. The experiment was concluded when the water exiting the glass tube turned clear. After the test, the mass of the uncleaned grouting machine was recorded as m_2_, that of the uncleaned glass tube as m_3_, and that of the cleaned empty tube as m_4_. The dynamic water retention ratio is given by Equation (1):

(1) $$\alpha =\frac{m_{3}-m_{4}}{m_{1}-m_{2}}\times 100\%$$

In this formula, *α* stands for the dynamic water retention ratio; m_1_ is the combined weight of the grouting machine and slurry before the experiment; m_2_ is the weight of the grouting machine along with the slurry left inside after the test; m_3_ is the weight of the borosilicate glass tube together with the slurry retained within it; m_4_ is the weight of the empty borosilicate glass tube.

#### 2.3.5. Microstructural Analysis

Specimens for microscopic analysis were prepared following the procedures given in “Microstructural Analysis Methods of Cement-based Materials” [36]. A tungsten-filament scanning electron microscope (SEM) from TESCAN (Brno, Czech Republic), model TESCAN VEGA GMS, was employed for observation. X-ray diffraction (XRD) patterns were collected on a Rigaku MiniFlex 600 diffractometer (RigakuCorporation, Tokyo, Japan). Thermogravimetric-differential thermogravimetric (TG-DTG) measurements were carried out using a TGA/DSC1 thermal analyzer from METTLER TOLEDO, Greifensee, Switzerland.

Specimens cured to the target ages (3 d, 7 d, 14 d, and 28 d) are taken and preliminarily crushed. Surface laitance and impurities are removed, and core samples that are homogeneous and crack-free are selected. The fragments are then immersed in isopropanol for 3 h to stop hydration, followed by drying at 40 °C for 3 h to eliminate the solvent and residual moisture. The dried fragments are manually crushed into blocks approximately 5 mm × 5 mm × 3 mm. Finally, the samples are sealed and stored for SEM examination.

For the X-ray diffraction (XRD) experiment, the sample is first evenly distributed into a blind-hole glass sample holder and pressed to increase its packing density. The holder is then placed into the X-ray diffractometer, and the measurement is performed by operating the computer software. After the XRD pattern is obtained, the corresponding analysis software is used to determine the phase composition of the hardened slurry body.

Thermogravimetric-differential thermogravimetric (TG-DTG): The thermal stability of the specimen is tested using a thermogravimetric analyzer (TGA) under a nitrogen atmosphere. An alumina crucible containing 8–15 mg of the sample was heated from ambient temperature to 1000 °C at a ramp rate of 10 °C/min under a nitrogen flow of 50 mL/min. The mass loss behavior of the sample during heating is analyzed through TG and DTG curves to characterize the decomposition characteristics of hydration products and the high-temperature stability.

All tests are conducted in triplicate. Results are expressed as mean ± standard deviation. Statistical comparisons between two groups are made using an independent-samples *t*-test. A *p*-value less than 0.05 is considered statistically significant.

## 3. Results and Discussion

### 3.1. MWCNTs

#### 3.1.1. Compressive Strength Test

**Figure 4:**
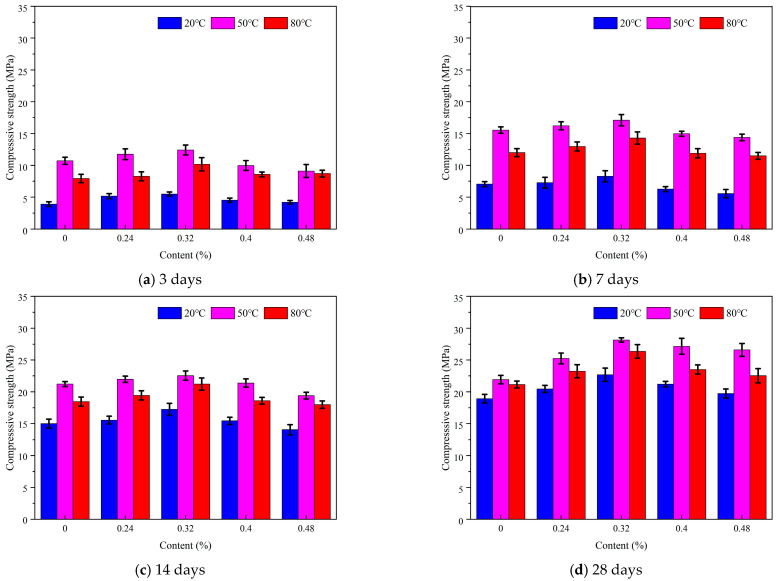
Compressive strength of hardened slurry with different MWCNTs contents. (**a**) 3 days; (**b**) 7 days; (**c**) 14 days; (**d**) 28 days.

The compressive strength values of hardened slurries with various MWCNT contents are presented in Figure 4 for curing temperatures of 20 °C, 50 °C, and 80 °C at ages of 3, 7, 14, and 28 days.

The compressive strength of hardened slurry increases first and then decreases with the rise in curing temperature. The strength at all curing ages reaches the maximum at 50 °C, and the strength of the group with 0.32% MWCNTs content exceeds 28 MPa at the age of 28 days. Although the strength at 80 °C is higher than that at room temperature of 20 °C, it is always lower than that at 50 °C. This indicates that a proper temperature facilitates the development of compressive strength of hardened slurry, while an excessively high temperature accelerates the early hydration of cement slurry and thus reduces the strength. The variation trend of compressive strength is consistent with that of C-F-S slurry reported by Zhang [12], proving that the addition of MWCNTs does not alter this rule. Meanwhile, MWCNTs exert no obvious influence on the age-dependent growth law of compressive strength of hardened slurry.

A comparison between the MWCNTs-added groups and the blank control group (0% content) shows that MWCNTs can enhance the strength development of hardened slurry at high temperatures. This reinforcing effect becomes more prominent as the temperature rises. The maximum strength increment of approximately 25% (6 MPa) is observed in the group with 0.32% MWCNTs at the curing temperature of 50 °C and the curing age of 28 days. Even under curing at 80 °C, the 28-day compressive strength of hardened slurry increases by about 19% (4 MPa). Obvious differences exist in the compressive strength among groups with different MWCNTs contents, especially for the 0.40% and 0.48% content groups. At 28 days, the group with 0.32% MWCNTs exhibits a distinct advantage in compressive strength. This phenomenon verifies the agglomeration effect of MWCNTs. An appropriate content of MWCNTs can effectively fill pores and bridge microcracks, whereas excessive addition causes MWCNTs to agglomerate and form stress concentration points, which in turn reduce the strength of hardened slurry. Nevertheless, the hardened slurry with excessive MWCNTs still achieves a higher 28-day compressive strength than the blank group. It indicates that the adverse effect of agglomeration-induced stress concentration on compressive strength is weaker than that of internal pores.

#### 3.1.2. Other Performances

**Figure 5:**
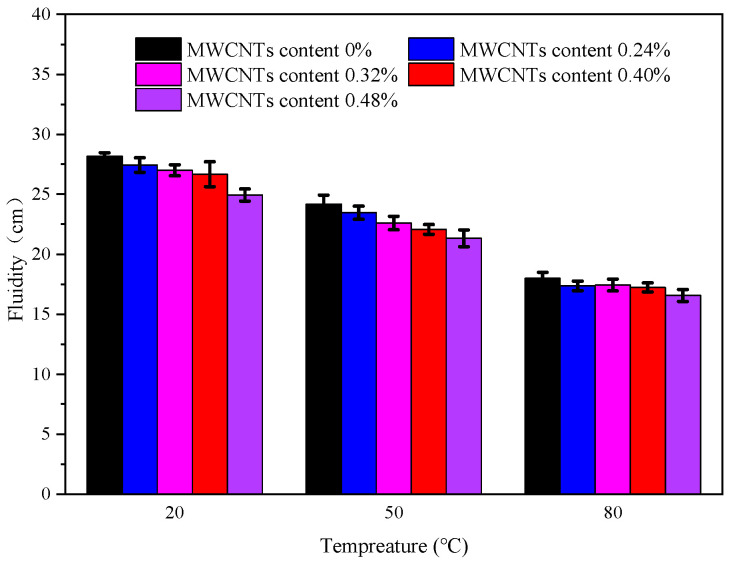
Fluidity of slurry with different MWCNTs contents.

Figure 5 presents the fluidity of slurry with different MWCNT contents cured at 20 °C, 50 °C, and 80 °C. It can be seen that temperature is a major factor affecting slurry fluidity, which decreases remarkably with increasing temperature. The fluidity ranges from 25 cm to 28 cm at 20 °C and drops to 21 cm to 24 cm at 50 °C, representing a reduction of approximately 14%. At 80 °C, the fluidity further declines to 16 cm-18 cm, with a decrease of around 25% compared with that at 50 °C, showing an evidently enlarged decline range. At a fixed temperature, the fluidity gradually decreases as the MWCNTs content increases, and the reduction becomes more significant at higher contents. For instance, at 20 °C, when the MWCNTs content rises from 0% to 0.40%, the fluidity only falls from 28 cm to 27 cm, a decrease of merely 3.6%. When the content increases from 0.40% to 0.48%, the fluidity drops from 27 cm to 24.8 cm with a reduction of 8.2%, which is a notable rise in decline amplitude. This is because excessive MWCNTs tend to agglomerate. The formed agglomerates increase inter-particle resistance and slurry viscosity, thus leading to a reduction in fluidity.

Overall, the addition of MWCNTs has an insignificant adverse effect on slurry fluidity. In terms of fluidity performance, the contents of 0.24%, 0.32% and 0.40% are all acceptable, as each reduces the slurry fluidity by no more than 6%.

**Figure 6:**
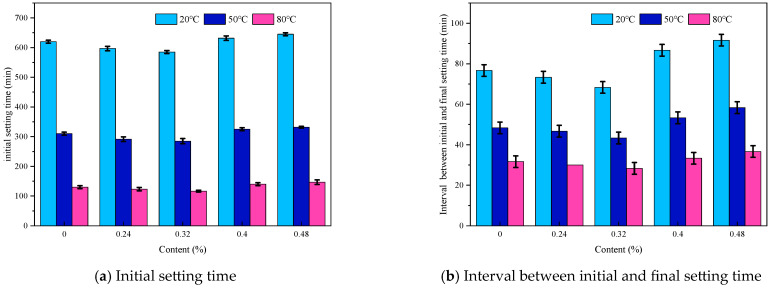
Setting time of slurry with different MWCNTs contents. (**a**) Initial setting time; (**b**)Interval between initial and final setting time.

The initial setting time (IST) and the time interval between initial and final setting (IAF) for slurries with different MWCNTs contents, cured at 20 °C, 50 °C, and 80 °C, are shown in Figure 6. It can be observed that for slurries with the same MWCNTs content, both the IST and IAF decrease significantly as the curing temperature rises. For instance, for the slurry without MWCNTs (0% content), the IST is approximately 620 min at 20 °C, which drops to around 310 min at 50 °C and merely 130 min at 80 °C. In terms of the IAF, when the MWCNTs content is 0.32%, the IAF is about 70 min at 20 °C, reduces to 45 min at 50 °C, and falls to roughly 30 min at 80 °C.

It is evident that the setting time of the slurry decreases first and then increases with the rising content of MWCNTs. When the MWCNTs content increases from 0% to 0.32%, the IST of the slurry is reduced by approximately 40 min (6.5%), and the IAF decreases by about 6 min (8.5%). As the content further rises from 0.32% to 0.48%, the initial setting time increases by around 70 min (12%), and the time gap between initial and final setting grows by roughly 18 min (25%). At low contents, MWCNTs shorten the setting time of the slurry. Due to their large specific surface area, MWCNTs act as nucleation sites for cement hydration, accelerating the dissolution of clinker minerals and the formation of hydration products, including C-S-H gel and ettringite, which consequently promotes the hydration process. In contrast, excessive MWCNTs tend to agglomerate and wrap around cement particles, retarding local hydration and leading to prolonged setting time with greater fluctuations. Excess MWCNTs exert a more pronounced adverse effect on the setting performance of the slurry, which should be avoided in practical engineering. Accordingly, the MWCNTs content is recommended to be no more than 0.32% from the perspective of setting time.

**Figure 7:**
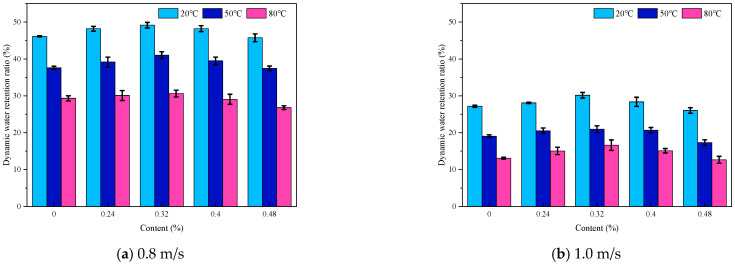
Dynamic water retention ratio of slurry with different MWCNTs contents. (**a**) 0.8 m/s; (**b**) 1.0 m/s.

Figure 7 illustrates the dynamic water retention ratio of slurries with different MWCNTs contents under flow velocities of 0.8 m/s and 1.0 m/s. At a flow velocity of 0.8 m/s, the retention ratio of all slurry groups increases initially with the addition of MWCNTs and then decreases, attaining its peak at a dosage of 0.32%. For slurries with the same MWCNTs content, the dynamic water retention ratio decreases obviously with the increase in curing temperature. Taking the slurry with 0.32% MWCNTs as an example, the retention ratio is approximately 49% at 20 °C, drops to around 41% at 50 °C, and falls to merely 31% at 80 °C. The slurry without MWCNTs (0% content) exhibits the lowest dynamic water retention ratio, while the maximum value is obtained at 0.32% MWCNTs content across all temperatures. This indicates that the addition of an appropriate amount of MWCNTs can effectively improve the anti-scouring performance of the slurry. When the flow velocity increases to 1.0 m/s, the dynamic water retention ratio under the same conditions decreases by an average of 10–15% compared with that at 0.8 m/s. For instance, for the slurry with 0.32% MWCNTs at 20 °C, the retention ratio decreases from about 49% to 30%. Although the retention ratio of all groups declines remarkably, its variation trend remains basically consistent with that at 0.8 m/s. This is mainly attributed to the fibrous network structure of MWCNTs, which builds a spatial skeleton inside the slurry, enhances structural stability and reduces the mass loss caused by water flow scouring.

It can be observed that the variation trend of the dynamic water retention ratio with MWCNTs content is basically consistent with that of the setting time, which increases first and then decreases, with the turning point occurring at the content of 0.32%. The incorporation of MWCNTs can enhance the cohesion between slurry particles and thereby reduce mass loss under flowing water. Nevertheless, when the MWCNTs content rises to 0.48%, the dynamic water retention ratio is roughly equivalent to that of the blank group. This phenomenon is probably caused by uneven internal cohesion induced by excessive MWCNTs.

#### 3.1.3. Microstructural Test

(1) XRD and TG-DTG test

**Figure 8:**
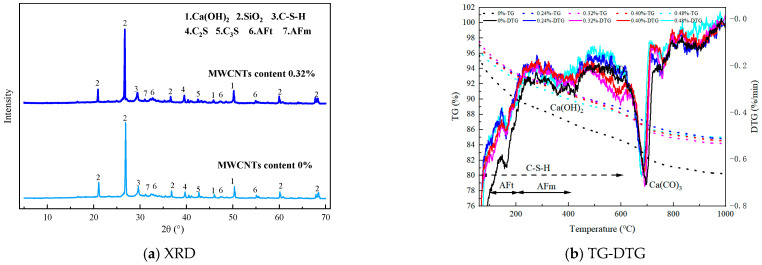
XRD and TG-DTG results of hardened slurry with different MWCNTs contents. (**a**) XRD; (**b**) TG-DTG.

Figure 8 presents the XRD patterns of hardened slurries with MWCNT contents of 0% and 0.32%, as well as the TG-DTG curves of specimens with MWCNT dosages of 0%, 0.24%, 0.32%, 0.40%, and 0.48%. All samples were cured at 80 °C for 28 days.

From the XRD quantitative analysis, the Ca(OH)_2_ content increased from 15.3 ± 0.5% to 16.6 ± 0.3% with the addition of 0.32% MWCNTs, and this difference was statistically significant (*p* = 0.0181). The C-S-H content decreased from 12.9 ± 0.5% to 11.7 ± 0.4% (*p* = 0.0315). The SiO_2_ peak content remained roughly unchanged between the two groups. The test results of hardened slurries with two different MWCNT contents are generally similar, with no obvious difference. Only the peak of AFt is 5.6% ± 0.2% in the 0% content group, which is slightly higher than 3.4% ± 0.1% in the 0.32% content group. According to the TG-DTG curves, the incorporation of MWCNTs reduces the mass loss of hardened slurries by approximately 3.5%. This indicates that more AFt is formed in the blank group. AFt contributes to the early strength of hardened slurry but impairs its late strength. Accordingly, MWCNTs can retard the formation of AFt in hardened C-F-S slurry at the later hydration stage and further improve the compressive strength of the hardened body, which is consistent with the aforementioned result that slurries containing MWCNTs exhibit higher compressive strength.

In TG-DTG tests, the thermal decomposition temperature ranges of the hydration products are roughly as follows: the thermal decomposition temperature of ettringite (AFt) is 100–200 °C; that of monosulfate (AFm) is 200–400 °C; that of calcium hydroxide (CH) is 400–500 °C; that of calcium silicate hydrate gel (C-S-H) is 50–600 °C; and that of calcium carbonate (CaCO_3_) is 600–800 °C.

The TG-DTG curves of hardened slurries with five different MWCNT contents show an overall similar trend with only minor discrepancies. At 600 °C, the hardened slurry with 0.24% MWCNTs presents the minimum mass loss of 9.39% among all groups, while its mass loss at 200 °C is 2.27%, which is close to the values of 2.48% for the 0.32% group and 2.38% for the 0.40% group. The contents of AFm and C-S-H gel in this group are 2.36% and 9.39%, both lower than those in the 0.32% and 0.40% groups. The slurry with 0.48% MWCNTs exhibits the maximum mass loss of 3.17% at 200 °C but reaches its minimum value at 1000 °C, and its contents of AFm and C-S-H gel are also lower than those of the other three groups. Since AFm and C-S-H gel are beneficial to the late strength of hardened slurry, the 0.32% and 0.40% groups deliver superior compressive strength, which agrees with the compressive strength test results presented in Section 3.1.1. In addition, the differences among the TG-DTG curves of all groups are marginal, demonstrating that MWCNTs exert a limited influence on the composition of hydration products of hardened slurry.

(2) SEM test

**Figure 9:**
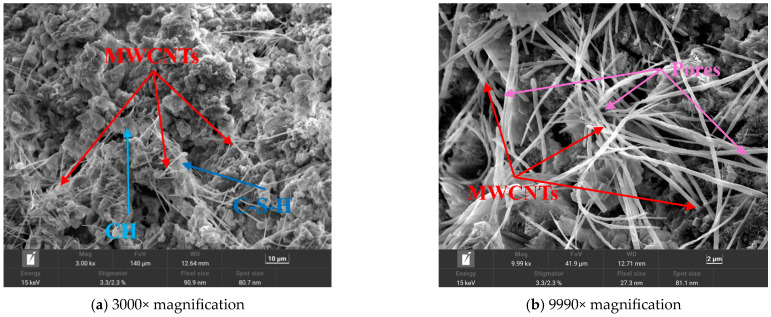
SEM pictures of hardened slurry incorporated with MWCNTs. (**a**) 3000× magnification; (**b**) 9990× magnification.

Figure 9 shows the SEM images of hardened C-F-S slurry with 0.32% MWCNTs cured at 80 °C for 28 days (magnification: 3000× for the left image and 9990× for the right one). Figure 9a at 3000× magnification reveals the overall distribution of cement hydration products, including platy calcium hydroxide crystals (CH) and flocculent calcium silicate hydrate (C-S-H) gel, as well as MWCNTs. In general, fibrous MWCNTs encapsulate various hydration products and partially interpenetrate between C-S-H gel and CH crystals to form a bridging structure. The results demonstrate the modification effect of MWCNTs on the microstructure of hardened cement slurry: encapsulation facilitates sufficient hydration reactions, while the bridging effect connects hydration product clusters, improves the overall strength, and remedies internal structural defects.

Figure 9b is the SEM image at 9990× magnification, where the microscopic characteristics of MWCNTs are clearly observed. The MWCNTs have a diameter of several tens of nanometers and a length of several micrometers, presenting a favorable aspect ratio. Some MWCNTs overlap with one another to form a network structure, which effectively fills the internal pores of the matrix (marked as Pores in Figure 9b) and agglomerates with hydration products, indicating excellent interfacial compatibility between MWCNTs and hydration products. It can also be seen that some hydration product clusters attach to MWCNTs, which strengthens the weak regions inside the hardened slurry and further enhances its overall strength. After incorporating MWCNTs, the internal pore structure of hardened C-F-S slurry is optimized. Meanwhile, the bridging effect restrains the initiation and propagation of microcracks, thereby improving the mechanical properties and durability of the hardened slurry.

### 3.2. PCE and HPMC

#### 3.2.1. Fluidity and Grout Retention Ratio Under Dynamic Water Test

**Figure 10:**
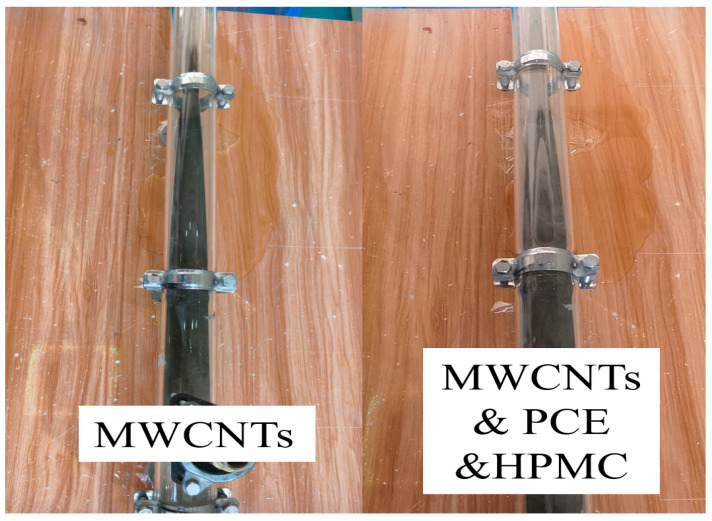
Retention state of different content in the slurry.

Figure 10 shows the retention state of slurries under flowing water. The left image presents the slurry incorporated with MWCNTs, while the right one corresponds to the slurry containing MWCNTs, PCE, and HPMC. It is observed that PCE and HPMC can effectively enhance the slurry’s dynamic water retention performance. After adding PCE and HPMC, the slurry can be retained over a longer distance, as reflected by its position closer to the water outlet in Figure 10, and the retained slurry volume also increases significantly.

**Figure 11:**
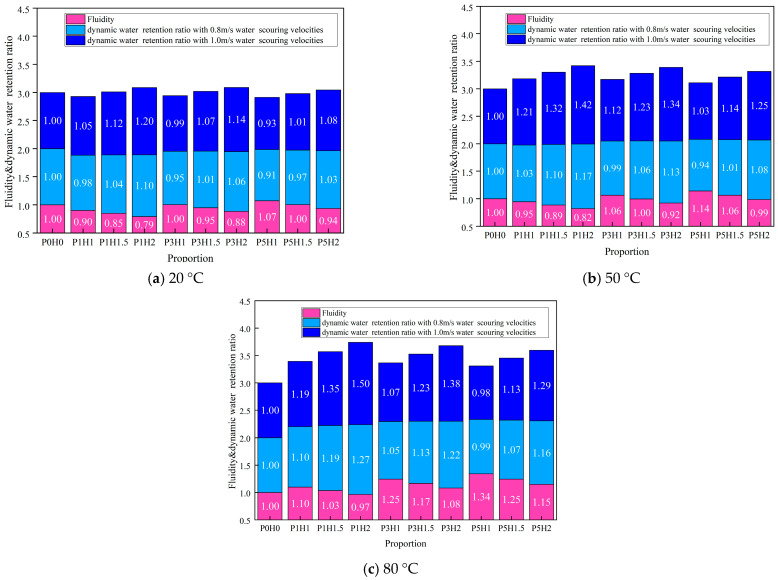
The fluidity and dynamic water retention ratio of slurry mixed with PCE and HPMC. (**a**) 20 °C; (**b**) 50 °C; (**c**) 80 °C.

To investigate the fluidity and dynamic water retention ratio of composite systems containing PCE and HPMC under different temperatures, tests were performed at 20 °C, 50 °C, and 80 °C. The group without PCE and HPMC (P0H0) was set as the blank group. An orthogonal test was carried out with PCE contents of 0.10%, 0.30%, and 0.50%, and HPMC contents of 0.10%, 0.15%, and 0.20%. The test results are shown in Figure 11. All data in Figure 11 are relative values, with the P0H0 group taken as the control group and its value defined as 1.00.

It is found that PCE exerts a positive effect on slurry fluidity, whereas HPMC plays a negative role. As the PCE content increases from 0.10% to 0.50%, the fluidity of groups P1H1, P3H1, and P5H1 rises gradually. The P5H1 group reaches the peak fluidity with a relative value of 1.07, which is higher than 0.90 for P1H1 and 1.00 for P3H1 at the same HPMC content. This demonstrates the dispersion and water-reducing effect of PCE, which effectively releases free water and improves slurry fluidity. At a fixed PCE content, fluidity decreases continuously as the HPMC content increases from 0.10% to 0.20%, as evidenced by the sequence P1H1 (0.90) > P1H1.5 (0.85) > P1H2 (0.79). This is due to the thickening and water-retaining effects of HPMC, which increase the viscosity of the slurry and hinder the movement of free water. At 20 °C, the combined addition of PCE and HPMC shows a limited improvement in fluidity, and most samples exhibit lower fluidity than the P0H0 group. However, the improvement becomes remarkable with the increase in temperature. Specifically, the relative fluidity of the P5H1 group reaches 1.34 at 80 °C, distinctly higher than 1.06 at 50 °C and 0.90 at 20 °C.

The results indicate that PCE can maintain acceptable fluidity of slurry under harsh conditions. At low temperatures, the original fluidity is relatively high, so PCE presents an unobvious enhancement effect. At high temperatures, slurry fluidity tends to decline sharply, while PCE releases immobilized water inside the slurry and improves flow performance [37].

Some researchers have proposed that the water film thickness is a key factor governing the fluidity of cement slurry when investigating the regulation mechanism of fluidity by superplasticizers [38,39,40,41]. Water in cementitious mixes can be categorized into filling water and excess water. The filling water occupies the voids between solid particles, whereas the excess water coats the particle surfaces with a water film, enhancing fluidity [42]. Carboxyl groups and other functional groups on the main chain of PCE molecules adsorb onto cement particle surfaces, rendering the particles negatively charged. The resultant electrostatic repulsion disperses the flocculated structure and releases the filling water trapped among particles. Meanwhile, polyoxymethylene groups on the PCE side chains extend into the slurry to form a steric hydration film, which prevents the re-agglomeration of cement particles [43,44]. These characteristics of PCE increase the content of excess water in the slurry, thereby enlarging the water film thickness and enhancing slurry fluidity.

Under flowing velocities of 0.8 m/s and 1.0 m/s, most groups with combined admixtures present higher dynamic water retention rates than the reference group P0H0 (relative value = 1.00). Given that PCE improves fluidity but reduces the dynamic water retention ratio, it is demonstrated that HPMC can effectively enhance the anti-scour performance of slurry, and such enhancement becomes more prominent as temperature rises. At the flow velocity of 0.8 m/s, groups P1H2, P3H2, and P5H2 exhibit distinctly higher dynamic water retention ratios than groups with lower HPMC dosages at the same PCE content. The increment is more remarkable at 1.0 m/s, where the maximum relative dynamic water retention rate reaches 1.50 for P1H2, corresponding to a 50% increase. Within the tested HPMC content range of 0.10–0.20%, the dynamic water retention ratio rises with increasing HPMC content. Nevertheless, it is noteworthy that P1H2 achieves the highest relative dynamic water retention ratio at 80 °C, accompanied by the lowest relative fluidity of 0.97, which is even lower than that of the P0H0 group.

During hydration, HPMC forms thin films among slurry particles, optimizes the structure of the interfacial transition zone, increases the bond strength between grout and substrate, and thus raises the overall viscosity of the slurry [45,46,47,48]. Hydroxyl and ether groups in HPMC bind with water molecules via hydrogen bonds. Free water is then encapsulated within HPMC molecular chains or immobilized on the chain surfaces [49,50]. This mechanism reduces slurry fluidity while improving its dynamic water retention capacity.

Although PCE impairs the slurry’s dynamic water retention performance and HPMC reduces fluidity, their combined effect can simultaneously improve both properties. Taking the fluidity and dynamic water retention performance at 80 °C as evaluation indices, the optimal combination is determined as P3H2. Despite the higher dynamic water retention ratio of P1H2, its relative fluidity of 0.97 is lower than that of the P0H0 group. Considering the inherently poor fluidity of slurry at 80 °C, mix proportions with fluidity below the P0H0 level are excluded. For P3H2, the relative fluidity is 1.08, and the relative dynamic water retention rates reach 1.22 and 1.38 under flow velocities of 0.8 m/s and 1.0 m/s, respectively. In addition, P5H1 is recommended when fluidity is prioritized, while P1H2 is the preferred option for maximum dynamic water retention.

#### 3.2.2. Other Performances

**Figure 12:**
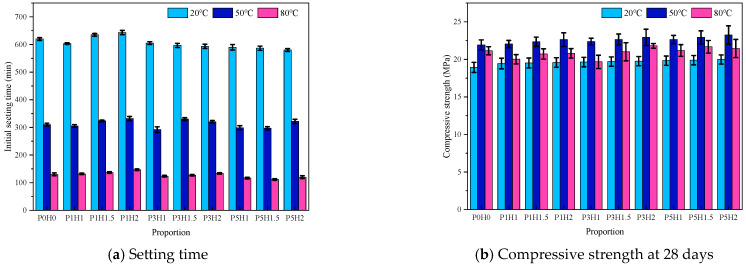
Initial setting time of slurry and compressive strength of hardened slurry. (**a**) Setting time; (**b**) compressive strength at 28 days.

While improving the fluidity and the slurry’s dynamic water retention performance, PCE and HPMC may affect other properties. Therefore, supplementary tests on setting time and compressive strength were conducted in this section. According to the test results, incorporating PCE and HPMC leads to minor changes in both the setting time of the slurry and the compressive strength of the hardened material, as shown in Figure 12, which displays the initial setting time and the 28-day compressive strength of the hardened specimens.

In terms of initial setting time, the values of ten groups of slurries are relatively concentrated at 20 °C, 50 °C and 80 °C, with a maximum difference of only approximately 27 min (4%) among groups. For compressive strength, the difference between the maximum and minimum values of hardened slurries cured at 80 °C for 28 days is about 1.8 MPa, with a relative deviation of roughly 7.8%. Although this deviation is small in absolute value, it remains within an acceptable range, considering that performance discreteness is inevitably induced by temperature fluctuation and differences in hydration progress under a high curing temperature of 80 °C. Furthermore, compared with the remarkable modification effects of C-F-S slurry and MWCNTs, which increase the compressive strength by about 50% and 25%, respectively, PCE and HPMC exert a far weaker influence on compressive strength. It can thus be concluded that PCE and HPMC have negligible impacts on the setting time and compressive strength of the slurry.

### 3.3. Limitations

Due to experimental constraints, the potential errors and limitations inherent in the test procedures of this study are analyzed as follows, laying the groundwork for future investigations:

(1) Certain methodological aspects of the high-temperature testing procedure may be subject to inadequacies, thereby potentially resulting in experimental deviations. Consequently, more appropriate testing protocols for evaluating the high-temperature performance of grouting materials require further investigation by subsequent researchers.
(2) Owing to limitations of the experimental setup, this study provides an insufficiently comprehensive characterization of the hydration mechanism of the grouting material. For example, quantitative analysis of SEM images for further characterization of pore features was not performed. Given the intricate and dynamic nature of the content and morphological changes in hydration products, continued in-depth research is warranted.
(3) This study only investigates the material at a curing age of 28 days and does not address its long-term durability. Accordingly, this aspect remains to be further explored and improved in future work.

## 4. Conclusions

This study investigated the effects of MWCNTs, PCE, and HPMC on the fluidity, dynamic water retention ratio, setting time, and compressive strength of C-F-S slurry by varying curing temperatures and curing ages. Meanwhile, XRD, TG, and SEM were adopted to explore the microscopic modification mechanism of MWCNTs, including the microstructure and hydration products of the materials. The main conclusions are summarized as follows:

(1) MWCNTs can remarkably improve the compressive strength of hardened slurry. It mainly inhibits the formation of ettringite inside hardened slurry at the late hydration stage and provides attachment and encapsulation sites for hydration products as well as bridges hydration product groups. The optimal MWCNTs content is determined as 0.32%. At this content, the compressive strength of hardened slurry cured at 80 °C for 28 days increases by approximately 20%.
(2) PCE improves the fluidity of the slurry, while HPMC enhances its dynamic water retention performance. The optimal combined content is 0.3% PCE and 0.2% HPMC. Under this proportion, the slurry fluidity rises by about 8% to around 17.7 cm. The dynamic water retention rate increases by roughly 22% and 38% under flow velocities of 0.8 m/s and 1.0 m/s, reaching 35.8% and 18.1%, respectively.

## Figures and Tables

**Table 1:** Chemical composition of raw materials.

Materials	Loss on Ignition	SiO_2_	Fe_2_O_3_	Al_2_O_3_	CaO	Others
Fly Ash	2.62%	49.1%	0.85%	42.8%	4.5%	0.13%
300-mesh silica	0.05%	99.76%	0.012%	0.13%	0.023%	0.025%
PO.42.5 R	0.56%	19.45%	4.42%	5.82%	63.31%	6.44%

Note: The data are provided by the supplier.

**Table 2:** Experimental scheme.

Test Content	Temperature	W/C Ratio	MWCNTs, PCE and HPMC	Other Test Variables
Fluidity	20 °C, 35 °C, 50 °C, 65 °C, 80 °C, 95 °C.	0.6	MWCNTs (0.24%~0.48%) PCE (0.10%~0.50%) HPMC (0.10%~0.20%)	/
Setting time				/
Compressive strength				Curing age (d): 3,7,14,28
Grout retention ratio under dynamic water				Scouring velocity in flowing water (0.8 m/s and 1.0 m/s)
SEM, XRD, and TG-DTG tests				Curing age (d): 3,7,14,28

## Data Availability

The original contributions presented in this study are included in the article. Further inquiries can be directed to the corresponding author.
